# Alkenylbenzenes in Foods: Aspects Impeding the Evaluation of Adverse Health Effects

**DOI:** 10.3390/foods10092139

**Published:** 2021-09-10

**Authors:** Andreas Eisenreich, Mario E. Götz, Benjamin Sachse, Bernhard H. Monien, Kristin Herrmann, Bernd Schäfer

**Affiliations:** 1 Department of Food Safety, German Federal Institute for Risk Assessment (BfR), Max-Dohrn-Str. 8-10, 10589 Berlin, Germany; mario.goetz@bfr.bund.de (M.E.G.); benjamin.sachse@bfr.bund.de (B.S.); bernhard.monien@bfr.bund.de (B.H.M.); bernd.schaefer@bfr.bund.de (B.S.); 2 Department of Pesticides Safety, German Federal Institute for Risk Assessment (BfR), Max-Dohrn-Str. 8-10, 10589 Berlin, Germany; kristin.herrmann@bfr.bund.de

**Keywords:** alkenylbenzenes, food, consumption, regulation, mixtures

## Abstract

Alkenylbenzenes are naturally occurring secondary plant metabolites, primarily present in different herbs and spices, such as basil or fennel seeds. Thus, alkenylbenzenes, such as safrole, methyleugenol, and estragole, can be found in different foods, whenever these herbs and spices (or extracts thereof) are used for food production. In particular, essential oils or other food products derived from the aforementioned herbs and spices, such as basil-containing pesto or plant food supplements, are often characterized by a high content of alkenylbenzenes. While safrole or methyleugenol are known to be genotoxic and carcinogenic, the toxicological relevance of other alkenylbenzenes (e.g., apiol) regarding human health remains widely unclear. In this review, we will briefly summarize and discuss the current knowledge and the uncertainties impeding a conclusive evaluation of adverse effects to human health possibly resulting from consumption of foods containing alkenylbenzenes, especially focusing on the genotoxic compounds, safrole, methyleugenol, and estragole.

## 1. Introduction

Alkenylbenzenes primarily occur as secondary plant metabolites in various herbs and spices (e.g., basil, fennel, and parsley) but are also present—albeit at lower levels—in agricultural crops, e.g., in tomatoes and apples [1,2]. Alkenylbenzenes are components of essential oils. Therefore, high concentrations can be found in food products made from aromatic parts of the abovementioned herbs and spices (e.g., fennel tea, basil-containing pesto, and plant food supplements) [3,4,5,6]. Since alkenylbenzenes have strong aromatic properties, they are also used as flavoring substances in foods and as fragrances in cosmetics [1]. Several alkenylbenzenes, such as safrole, methyleugenol, and estragole, are known to be toxic, and the most relevant toxicological endpoints include genotoxicity and carcinogenicity, whereby the toxicity is not caused by the parent compounds themselves but by their highly reactive metabolites [1].

The toxicity of alkenylbenzenes—especially their genotoxic and carcinogenic potential—is a controversially debated issue. Results of various toxicological studies demonstrated that single alkenylbenzenes, such as safrole, methyleugenol, and estragole, cause—amongst other things—genotoxic and carcinogenic effects in animal studies [1,7,8]. However, some other alkenylbenzenes, such as elemicin and apiol, have not yet been sufficiently assessed regarding their genotoxic and carcinogenic properties.

Beside the toxicity of single compounds, it has to be kept in mind that different foods may contain more than one alkenylbenzene, such as basil, which contains methyleugenol, estragole, and other compounds [9]. This is of particular importance for substances exhibiting a similar mode of action, since it may result in additive toxicity [9]. On the other hand, it was shown in different studies that the genotoxic potential of alkenylbenzenes may be reduced by other plant components, such as the sulfotransferase (SULT) inhibitor nevadensin [10,11]. This was called the matrix-derived combination effect [10]. However, the relevance of this effect in different food matrices is still an intensively discussed issue [12,13].

Occurrence data of alkenylbenzenes in different foods are necessary to assess human exposure. However, there are significant variations in occurrence levels, depending on, e.g., the analyzed samples (parts of plants, time of harvesting, region of origin), methodology (i.e., not standardized sample preparation and analytical methods), etc. Due to these differences, it is often not possible to assess occurrence data of different origins in a comparative manner, which complicates conducting a reliable exposure assessment.

Moreover, structural differences in alkenylbenzenes, such as in estragole vs. *trans*-anethole (see Table 1) influence toxicokinetics of these compounds. This, in turn, also affects the toxic (especially the genotoxic) potential of different alkenylbenzenes, which has to be taken into account for an assessment of the risks possibly resulting from exposure to these substances.

In the following parts, we will briefly summarize and discuss the current knowledge and the uncertainties impeding a reliable evaluation of the health risks resulting from alkenylbenzene exposure, especially focusing on the genotoxic compounds, safrole, methyleugenol and estragole. Moreover, we will shed some more light on ongoing discussions (e.g., the toxic potential of single compounds and mixtures) and some strengths as well as weaknesses of current experimental and analytical strategies regarding the risks possibly resulting from exposure to alkenylbenzenes in general.

## 2. Current Knowledge

### 2.1. Occurrence of Alkenylbenzenes

Consumers need to know which of their food consumption habits might result in high intake levels of genotoxic alkenylbenzenes in order to become able to draw informed decisions, whether to choose or not to choose a certain alkenylbenzene-containing aromatized or natural food.

#### 2.1.1. Alkenylbenzenes in Herbs and Spices

In the following part, some examples are described to shed more light on the complexity of alkenylbenzene composition in different herbs and spices.

##### Fennel

The herb fennel (*Foeniculum vulgare* Mill., Umbelliferae or Apiaceae) is cultivated in many countries all over the world. Essential oils can be obtained by steam distillation of the dried ripe fruits or other parts of the plant such as leaves, stems, or roots, as described by Trenkle [24]. The wild common fennel is bitter (var. *vulgare*), and the cultivated one is rather sweet (var. *dulce*). Essential oil yields can be 2–6%, the major constituent of which is usually *trans*-anethole (60–90%) [35,36]. Depending on the extraction methods used, estragole contents vary between 3.3–5.3% in the aerial parts of the plant [37]. Trenkle, in 1972 in the aerial parts of the sweet fennel, found (stems, leaves, and seeds) *trans*-anethole (9.7–54.7%), *cis*-anethole (0.1–0.8%), and estragole (2.0–3.0%) but no myristicin [24]. However, the fennel roots contained neither anetholes nor estragole but contained instead dill-apiol (45.6–62.7%), myristicin (2.5–10%), and parsley apiol (0.2%). The oil from sweet fennel fruits is used as a flavor component in many products. Hydrodistillation of fennel fruits may yield up to 88% estragole [38]. Very common in, e.g., Europe is the consumption of fennel tea infusions. The determination of estragole in infusions from different widely used commercial herbal teas based on *Foeniculum vulgare* seeds by an optimized headspace solid-phase microextraction followed by gas chromatography–mass spectrometry (GC–MS) analysis revealed levels of estragole to range within 50–250 µg/L [39] or even reach levels from 241–2058 µg/L in teas from teabags [40]. In preparations of tea extracts from herbal tea mixtures (*n* = 16) of the fennel–anise–caraway type, estragole contents ranged from 4.0–76.7 µg/L, whilst *trans*-anethole concentrations ranged from 83.2–7266.4 µg/L [41]. Interestingly, one hour following ingestion of fennel–anise–caraway tea by breastfeeding women, approximately 1% (i.e., 0.13 µg/L milk) of the consumed estragole via tea ingestion and up to 5% (i.e., 4.23 µg/L milk) of *trans*-anethole consumed via tea ingestion was found in the human milk of lactating mothers [41]. An earlier study could identify in breast milk from breastfeeding women, at the time point of two hours following ingestion of a 100 mg *trans*-anethole containing capsule, a mean concentration of 9.9 µg *trans*-anethole per liter of human breast milk. Peak concentrations of *trans*-anethole were 23.2 µg/L milk [42]. These results indicate that some alkenylbenzenes may even, as parent compounds, escape maternal hepatic metabolism and can be transferred into breast milk, albeit at very low concentrations. To our knowledge, other systematic studies that investigated metabolites of estragole and *trans*-anethole and other alkenylbenzenes in human breast milk are missing.

##### Basil

Sweet basil herb (*Ocimum basilicum* L., Labiatae or Lamiaceae) is, nowadays, cultivated in many countries around the world, originating probably from Africa and tropical Asia. The essential oil is generated from dried leaves and stems (aerial parts of the plant) by steam distillation. In a systematic study of essential oils obtained from the aerial parts of seven varieties of *Ocimum basilicum*, it was found that basil oils may contain, relative to other identified components, high amounts of methyleugenol (9.27–87.04%) and estragole (0–48.28%; only in the varieties “Lettuce Leaf” and “Dark Green”). The alkenylbenzene content depends on the basil variety, season, and the environmental conditions, as well as the maturation state at harvest, such as growth height. Another alkenylbenzene found in nearly all sweet basil oils investigated is eugenol (0–33.5%). All the studied varieties of *Ocimum basilicum*, except “Lettuce Leaf” (lowest contents of methyleugenol 9.24–15.45%), were very rich in methyleugenol (up to 87.04%) with dependence on solar irradiance, temperature and relative humidity as determining factors [25]. Earlier studies on the chemical components of *Ocimum basilicum* plants focused on the age and the leaf position at the stem [26], as well as differentiated the essential oil analysis derived from the flowers, leaves, and stems [27]. Eugenol levels were slightly higher in younger leaves, and methyleugenol levels predominated in older leaves, but appears to be more affected by leaf position. The flowers of basil collected in Turkey contained 58.26% estragole, 0.23% *trans*-anethole, and only 0.03% methyleugenol. The respective leaves contained 52.60% estragole, 0.55% *trans*-anethole, and 0.18% methyleugenol. Interestingly, the basil stems contained less estragole (15.91%), *trans*-anethole (0.10%) and methyleugenol (0.06%), but in addition, and exclusively found in stems, were dill-apiol (50.07%), apiol (9.48%), elemicin (0.30%), and low amounts of eugenol (0.12%). There still appears to be no full clarity on the biosynthetic pathways of alkenylbenzenes in basil species and the environmental factors influencing the expression of biosynthetic enzymes. As discussed by Vani and colleagues, chavicol *O*-methyltransferase identified in crude protein extracts of sweet basil may be responsible for the conversion of chavicol to estragole [28]. Eugenol may be transformed into methyleugenol by eugenol *O*-methyltransferase, both enzymes most likely use *S*-adenosylmethionine (SAM) as the methyl donor. However, formation of estragole and methyleugenol is strongly dependent on season and on solar irradiance. Estragole contents may even reach 81% if leaves of *Ocimum basilicum* are extracted with n-hexane before analysis with GC–MS [28]. Using the same techniques, Vani et al. identified high contents of methyleugenol (36–76%) in n-hexane extracts of another basil species *Ocimum tenuiflorum* (also named *Ocimum sanctum*), mainly grown in India.

Exposure to estragole and methyleugenol might be low at common use levels of fresh basil, but there are only a few systematic investigations of alkenylbenzene contents in food preparations of various recipes. Moreover, with consumption of an essential oil merchandised as a food supplement or the plants being part of dishes in which basil is prepared together with other culinary oils, consumer exposure to alkenylbenzenes may increase considerably. An example is given by Bousova and colleagues who found estragole at a high concentration of 101 mg/kg pesto product [43]. This traditional dish from Genova, Italy, mainly consists of olive oil, hard cheese, pine nuts, garlic, salt, and basil leaves. Very varying levels of estragole in pesto preparations have been reported (0.05–19.30 mg/kg versus fresh basil containing 10.21–16.05 mg/kg [44]. Another study reported levels of estragole in “Pesto Genovese” (3.2–34.1 mg/kg estragole) [6]. The same study additionally reported levels of methyleugenol (22.9–56.4 mg/kg) and even myristicin (13.2–15.8 mg/kg), and in one sample apiol (3.4 mg/kg).

Recently, Sestili et al. concluded that maximum level should be precautionarily defined for alkenylbenzenes from different basil species and thus different chemotypes that contain high amounts of methyleugenol and estragole in essential oils intended for consumption with food [45]. Currently, no precise data regarding the consumption of basil or the realistic levels of different alkenylbenzenes in this herb is available.

Further occurrence data of alkenylbenzenes in other herbs and spices, such as allspice, anise, and tarragon, are summarized in Table 1.

#### 2.1.2. Alkenylbenzenes in Aromatized and Fortified Food Products

Many essential oils contain alkenylbenzenes. The most prominent examples of essential oils used in food and beverages are oils produced from basil, fennel, tarragon, parsley, anise, star anise, nutmeg, and mace [46]. Such oils are mostly obtained from plant components by hydrodistillation, steam distillation, solvent extraction, supercritical fluid extraction, ultrasound- or microwave-assisted extractions, or a combination of diverse techniques [47].

When consumed with food products, essential oils can contribute significantly to the overall exposure to potentially genotoxic and carcinogenic compounds. These oils usually contain between 30–90 weight% of the critical ingredient. Depending on the amount of essential oils added to processed foods for reasons of flavoring or food supplements, unknown amounts of alkenylbenzenes exist as undefined mixtures in finished food products. Although these oils are generally meant to be used in very small volumes to refine culinary products, it is, however, difficult to calculate people’s overall exposure, also because of the individual food intake habits. As a special case, plant food supplements may contain high amounts of essential oils.

##### Essential Oils Used as Food Flavorings

Depending on the origin of the plants, basil oils and tarragon oils contain variable but very high amounts of estragole (methylchavicol). Whilst basil oils may be widely used by consumers, tarragon oils are mainly used for food aroma compositions [32]. Parsley seed oils are used for seasonings for meat and sauces. They contain apiol, myristicin, and 2,3,4,5-tetramethoxy-allylbenzene. Pimento oils from berries or leaves of that tree predominantly contain eugenol and can also be used for food aroma compositions. In most essential oils containing anethole, the *trans*-anethole isomer by far predominates the *cis* isomer. *trans*-Anethole contents are high in fennel, anise, and star anise oils [46].

Nutmeg oils and mace oils are mainly used for cola-flavored soft drinks and may contain myristicin and other alkenylbenzenes. Thus, it is expected that all the ingredients of nutmeg are part of cola-flavored soft drinks to various extents. Major compounds of nutmeg and mace oils are sabinene, *alpha*- and *beta*-pinene, myrcene, limonene, and at least five different alkenylbenzenes. Myristicin, safrole, and elemicin determine the flavor of these oils to a great extent. Myristicin, safrole, elemicin, methyleugenol, and eugenol could be quantified in cola-flavored soft drinks [48]. However, an at least 30-fold variation in the levels of safrole and myristicin, for example, has been reported in different nutmeg oils of specific geographical origins, ranging from 0.1–3.2% and from 0.5–13.5%, respectively [48]. Consequently, the amounts of safrole and myristicin were quantified in cola-flavored soft drinks of different brands and following different processing procedures, including various storage conditions. Variation in the contents of safrole and myristicin in different cola-flavored soft drinks were identified to be approximately two to three orders of magnitude [49]. Minimum contents of safrole and myristicin were 0.6–0.4 µg/L, and maximum levels ranged from 43.9–325.6 µg/L for safrole and myristicin, respectively. Other alkenylbenzenes than safrole and myristicin were not evaluated in those cola-flavored soft drinks, so that the total content of alkenylbenzenes in cola-flavored soft drinks remains to be elucidated.

### 2.2. Toxicity of Alkenylbenzenes

#### 2.2.1. Toxicokinetic Impact on Toxic Properties of Alkenylbenzenes

**Figure 1:**
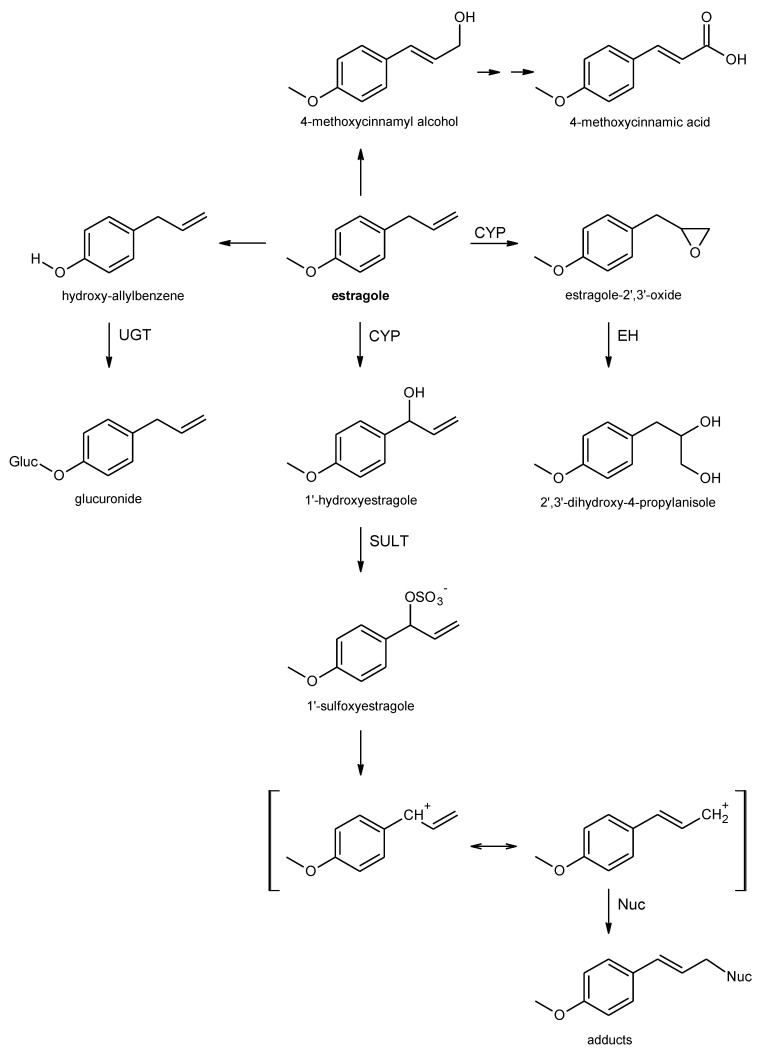
Important metabolic steps of estragole as an example for the allylalkoxybenzenes. CYP, cytochrome P450-monooxygenase; UGT, uridine 5′-diphospho-glucuronosyltransferase; EH, epoxide hydrolase; SULT, sulfotransferase; Nuc, nucleophile (e.g., DNA, protein).

Following oral exposure, alkenyl benzenes are rapidly absorbed from the gastrointestinal tract. The low systemic bioavailability of the ingested parent compounds, however, points to a pronounced first pass metabolism [50,51,52,53,54,55,56,57,58]. Different metabolic routes have been observed for alkenylbenzenes, resulting either in bioactivation (toxification) or in detoxification of the parent compounds. The extent of the different pathways depends on species and dose [59,60]. Important metabolic steps of estragole as an example for the alkoxyallylbenzenes are shown in Figure 1.

For alkoxyallylbenzenes, such as safrole, methyleugenol, and estragole, metabolic pathways include *O*-dealkylation of the alkoxy substituents at the aromatic ring, epoxidation at the double bond of the allylic side chain, and 1′-hydroxylation of the allylic side chain [59,60]. *O*-dealkylation of an aromatic alkoxy group (or demethylenation) leads to the formation of the corresponding phenolic (catecholic) derivatives [50,61,62,63,64,65,66,67]. The resulting phenol group can be further metabolized via phase II enzymes to stable glucuronides or sulfate conjugates that are rapidly excreted in the urine [54,59]. Therefore, this metabolic route can be considered a detoxifying pathway.

Epoxide formation at the double bond of the allylic side chain represents another metabolic route. Following epoxidation, the epoxide ring can be cleaved by epoxide hydrolases to form diols. The occurrence of 2′,3′-dihydrodiols (and sometimes the epoxides) in urine of rodents treated with different alkenylbenzenes points to the formation of these metabolites in vivo [64,67,68]. Additionally, detoxification of epoxides by glutathione-*S*-transferases was observed [69,70]. If detoxification does not occur fast enough and/or to a critical extent, epoxides may be attacked by nucleophilic structures of the cell. Experiments of Guenthner and Luo have demonstrated that the epoxides are capable forming covalent adducts with proteins and DNA in vitro, suggesting a potential for genotoxicity [69,71]. However, the toxicological relevance of that pathway is generally considered low, since the epoxide is rapidly detoxified by epoxide hydrolases or via glutathione conjugation, with humans generally having a higher epoxide hydrolase activity than rats [59,60,69].

The first step of the third pathway is the cytochrome P450 (CYP)-mediated 1′-hydroxylation of the allylic side chain [59,60]. 1′-hyroxy derivatives were detected as metabolites in the urine of rodents and humans following oral exposure to alkenylbenzenes [50,57,58,64,68]. On one hand, 1′-hydroxy derivatives can be further metabolized by glucuronidation, leading to detoxification, as demonstrated for 1′-hydroxyestragole [72]. Another option for detoxification, especially in humans, is the oxidation to the corresponding oxo derivative, which may be conjugated with glutathione [73,74,75]. However, bioactivation is also possible as the 1′-hydroxy alkenylbenzenes can subsequently be sulfoconjugated by SULTs. The resulting allylic sulfate esters are instable and may react with cellular nucleophiles, such as proteins or DNA [76,77]. This metabolic pathway is considered primarily responsible for the tumorigenic activity of some allylalkoxybenzenes, such as safrole, estragole, and methyleugenol [8,78,79]. The toxicological relevance of this pathway is also underlined by the finding that co-administration of the SULT-inhibitor pentachlorophenol (PCP) drastically reduced the carcinogenic activity of safrole in rodents [80]. Apart from that, the reactive sulfate esters may by detoxified by glutathione conjugation, yielding mercapturic acid derivatives. Indeed, the occurrence of *N*-acetyl-*S*-[3′-(4-methoxyphenyl)allyl]-L-cysteine—the mercapturic acid formed from 1′-sulfoxy estragole—has been detected in the urine of human volunteers after drinking fennel tea containing approximately 2 mg estragole [52]. Of note, although the highly reactive metabolites are formed via 1′-hydroxylation followed by sulfoconjugation at 1′-position, the final adducts are formed at the sterically less hindered 3′-position [52,77,81].

In addition, modifications at the side chain, yielding the 3′-hydroxylated isomers with the double bond in 1′,2′-position, is also possible. Such metabolites, as well as the parent compounds, may undergo further conversion to 3′-hydroxy and 3′-oxo derivatives via different chemical reactions [61,81].

Whereas the detoxifying *O*-dealkylation appears to be predominant at relatively low dose levels in rodents, the fraction of 1′-hydroxylation at the allylic side chain—leading to the proximate carcinogenic metabolite—seems to increase at higher doses in rodent studies [68,82,83]. However, the formation of 1′-hydroxy metabolites is also possible at relevant dose levels in humans, as the 1′-hydroxy metabolite of estragole has already been detected in the urine of human volunteers after drinking fennel tea [58]. Likewise, the occurrence of *N*-acetyl-*S*-[3′-(4-methoxyphenyl)allyl]-L-cysteine—the mercapturic acid formed from 1′-sulfoxy estragole—in the urine of human volunteers after drinking fennel tea [52], as well as the detection of DNA adducts of 1′-sulfoxymethyleugenol in human liver samples [84,85], underlines the formation of reactive cations in humans. Of note, interindividual human variations, such as polymorphisms and lifestyle factors influencing the activity of certain enzymes involved in the metabolism of allylalkoxybenzenes, may also influence the level of bioactivation of these compounds [85,86,87,88]. To illustrate this, Tremmel et al. have shown that the number of methyleugenol-derived DNA adducts in human liver samples is associated with the SULT1A1 copy number polymorphism [85].

In contrast to safrole, methyleugenol, and estragole, no carcinogenic effects have yet been observed for other members of the alkoxyallylbenzenes, such as elemicin and apiol [79]. Of note, the available studies generally do not meet today’s standards for carcinogenicity studies, e.g., study duration was often too short. Results from physiologically based biokinetic modeling studies, however, suggest that the extent of bioactivation to the ultimate carcinogenic 1′-sulfoxy metabolites is in the same order of magnitude for safrole, methyleugenol, estragole, elemicin, and myristicin, also pointing to a toxicological relevance for the latter two compounds [74,89,90,91].

Alkoxyprop-1-enylbenzenes, such as *trans*-anethole, are generally considered less toxic compared to the alkoxyallylbenzenes [60], although *trans*-anethole also acts as a liver carcinogen at high dose levels [92]. This class undergoes similar metabolic changes, such as the alkoxyallylbenzenes [57,65,66,67,93,94]. However, formation of the 1′,2′-epoxide is assumed to be primarily responsible for the hepatotoxic effects of *trans*-anethole observed in rodent studies at higher dose levels [95]. The efficient detoxification by epoxide hydrolases and glutathione—as described for the alkoxyallylbenzenes—may limit the toxicological relevance at low exposure levels. Another major metabolic pathway for this class is hydroxylation at the 3′-position at the propenyl side chain. Interestingly, in contrast to the 1′-hydroxy metabolites formed from the alkoxyallylbenzenes, the 3′-metabolites of the alkoxyprop-1-enylbenzenes are not efficiently metabolized by SULTs but mainly undergo oxidative side chain modification, yielding alkoxy cinnamoyl derivatives and alkoxy benzoic acid derivatives that are further conjugated with glycine [51,57,62,66]. Nevertheless, it has recently been demonstrated that both *trans*-anethole and estragole may principally lead to the formation of the same DNA adducts and hemoglobin adducts at the 3′-position, although adduct formation resulting from *trans*-anethole is much lower. This adduct formation by *trans*-anethole observed in hepatic S9-mix was efficiently blocked by PCP, indicating that some of the primarily formed 3′-hydroxyanethole is also converted by SULTs into the reactive 3′-sulfoxyanethole [76].

No carcinogenicity has been observed for eugenol, a hydroxyallylbenzene [79,96]. For this structural class, the free phenolic hydroxyl group enables a rapid phase II conjugation, leading to hydrophilic and non-toxic metabolites that are subject to fast renal elimination [54]. This difference may explain the lower toxicity of hydroxyallylbenzenes compared to alkoxyallylbenzenes. Further metabolic routes exist for hydoxyallylbenzenes, e.g., isomerization of the double bond and quinone methide formation [59,60]. However, these pathways shall not be described here, as hydroxyallylbenzenes are not in the primary focus of this review.

For the hydroxyprop-1-enylbenzene derivative isoeugenol, evidence of carcinogenicity was observed in a two year study [97]. However, the relevance of these findings is not yet fully clear [60]. Generally, this class of compounds may undergo similar metabolic changes, similar to those of the other alkenylbenzenes. However, the combination of the free phenol group and the double bond in 1′,2′-position facilitates rapid detoxifying metabolism via phase II conjugation at the phenolic hydroxyl group and hydroxylation at 3′-positions [53].

#### 2.2.2. Aspects Regarding Genotoxic and Carcinogenic Effects of Alkenylbenzenes

##### Safrole

Regarding the toxicity of safrole, animal studies showed that administration led to the induction of tumors, e.g., in mice and rats [98]. In the early 1960s, the first data were published indicating that safrole causes carcinogenic effects in rat liver [99]. In the following years, results of different animal studies (e.g., in rat and mice) confirmed that safrole is a carcinogen in the liver and other tissues, such as the lung [100,101]. Moreover, it was demonstrated that this carcinogenic effect was—at least in parts—mediated via active metabolites, such as 1′-hydroxysafrole or, rather, 1′-sulfoxysafrole [80,98,101,102,103]. The mutagenic effect of safrole and its metabolites was also verified in vitro and in vivo [7,104]. The toxicological relevance of the genotoxic 1′-sulfoxysafrole is underlined by the finding that co-administration of the SULT-inhibitor PCP drastically reduced the carcinogenic activity of safrole in rodents [80]. Therefore, the Scientific Committee on Food (SCF) of the European Commission (EC) considered safrole as a genotoxic carcinogen in 2002 [98]. In line with this, International Agency for Research on Cancer (IARC) also classified safrole as “possibly carcinogenic to humans” (Group 2B) [105].

##### Estragole

Results of different in vivo studies indicated that treatment of mice with estragole or its metabolite 1′-hydroxyestragole led to the induction of hepatic tumors [8,79,106]. Results of further studies conducted in bacteria and in cell culture indicated that mutagenic effects were more pronounced following treatment with the metabolite 1′-hydroxyestragole than with the parent compound estragole [7,107]. Therefore, induction of liver tumors seems to depend on the formation of 1′-hydroxymetabolites [8,31] that are further activated to highly reactive 1′-sulfoxy metabolites [108]. Based on the available data, the SCF of the EC concluded that estragole is genotoxic and carcinogenic [109]. Therefore, it was not possible to establish a safe exposure limit, and usage restrictions were recommended [109].

##### Methyleugenol

Long-term studies have revealed that methyleugenol induces liver and neuroendocrine tumors in rodents [79,110]. In this context, the National Toxicology Program (NTP) stated that there was clear evidence for carcinogenic activity in rats and in mice [110]. Methyleugenol was considered to be a multisite and multispecies carcinogen [111]. Different in vitro studies provided inconclusive results regarding mutagenicity of methyleugenol. In bacterial test systems, no mutagenic activity of methyleugenol was found without metabolic activation, whereas, e.g., in mammalian cell culture, a genotoxic activity was observed [110,112,113,114]. Moreover, the 1′-hydroxy- and 2′,3′-epoxy-metabolites were also found to be mutagenic in vitro [112,114]. In 2000, de Vincenzi et al. concluded from these findings that methyleugenol is a naturally occurring genotoxic carcinogen, exhibiting a DNA-binding potency similar to that of safrole. In line with this, the SCF of the EC also stated that methyleugenol has been demonstrated to be genotoxic and carcinogenic and recommended reduction in exposure and restrictions in use levels for this substance [111]. Substantiating this, IARC classified methyleugenol in 2013 as “possibly carcinogenic to humans” (Group 2B) [115].

##### Other Alkenylbenzenes

Even if some alkenylbenzenes are structurally closely related, such as estragole and *trans*-anethole or eugenol and methyleugenol (see Table 1), they often exhibit a different genotoxic and carcinogenic potential.

For eugenol, long-term studies revealed no mutagenic potential and no carcinogenic effects in rodents [79,96,116]. Based on the available data and a corresponding quality assessment, different authorities considered eugenol as not genotoxic or carcinogenic [117,118].

In contrast to eugenol, isoeugenol was found to exhibit carcinogenic activity in rodents (e.g., in the liver of mice [97,119]). The relevance of this finding, however, is not fully clear, yet [60]. Results of various in vitro and in vivo studies showed no mutagenic activity [113,120], whereas some few studies indicated a potential genotoxic activity at higher concentration in vitro [121]. However, based on the available data, isoeugenol was considered to be a non-genotoxic carcinogen by Joint Food and Agriculture Organization/ World Health Organization (FAO/WHO) Expert Committee on Food Additives (JECFA) and European Food Safety Authority (EFSA). Moreover, it was concluded that isoeugenol would not rise a safety concern at the estimated intake levels arising from use as a flavoring substance [122,123,124].

In chronic rodent studies, *trans*-anethole did not increase the tumor incidence [79,92,95]. Moreover, most studies performed on the mutagenicity of *trans*-anethole failed to show a mutagenic activity, whereas only a few studies—often offering a limited reliability or reproducibility—indicated a mutagenic activity [7,95,125,126]. Based on the available data, JECFA concluded that *trans*-anethole is not genotoxic [126]. Due to the hepatotoxic effects observed in rats (considered as secondary to its cytotoxic properties and possibly mediated via the anethole epoxide), safety concerns were formulated by JECFA regarding the use of *trans*-anethole as flavoring agent [126]. In this context, recently published results have to be mentioned, showing that both *trans*-anethole as well as the structurally related estragole are able to form DNA and hemoglobin adducts, even if the adduct formation resulting from *trans*-anethole is much lower [76].

#### 2.2.3. Toxicity of Alkenylbenzenes from Complex Food Matrices

In 2019, EFSA published a guideline document regarding the genotoxicity assessment of chemical mixtures [127]. In this guideline, EFSA recommends the application of a component-based approach to chemical mixtures, in which the genotoxic potential of all components are assessed individually. Consequently, this means that, if a mixture contains one or more chemical substances that are individually assessed to be genotoxic (in vivo via a relevant route of administration), the whole mixture raises concern for genotoxicity [127]. In line with this, the toxicity of alkenylbenzenes is, in most cases, tested in vivo only with pure substances [128].

In 2008, Rietjens and colleagues mentioned that exposure of consumers to alkenylbenzenes under everyday conditions most often occurs in presence of a “normal” food matrix, such as herbs (e.g., basil) or in processed food products (e.g., pesto sauces or beverages), respectively [4,128]. In this context, it has to be kept in mind that—due to the presence of several alkenylbenzenes in most foods/matrices, such as anise or basil—additive effects must be assumed regarding the genotoxicity of mixtures. In line with this, Dusemund et al. stated that specific foods may contain more than one alkenylbenzene [9]. This, in turn, could possibly lead to additive or combined toxicity effects of different alkenylbenzenes taken up via the same food. Moreover, other researchers have also concluded that the consumption of food containing different alkenylbenzenes may contribute to combined toxic effects [3,6,129,130]. This may also apply to other compounds present in a distinct food matrix (e.g., contaminants), which can affect similar endpoints like alkenylbenzenes, such as genotoxicity by a comparable or different mode of action [131,132,133].

On the other hand, Rietjens et al. stated that certain food matrices may also reduce the genotoxic potential of alkenylbenzenes via interaction on a metabolic level [128]. Bioactivation of alkenylbenzenes, such as safrole and estragole, plays an important role for mediating their genotoxic effects via the generation of proximate (1′-hydroxy metabolites) and ultimate (1′-sulfoxy metabolites) carcinogenic intermediates [89,102,128].

In 2008, Jeurissen et al. found that methanolic basil extract reduces the genotoxic effect of 1′-hydroxyestragole in vitro via inhibition of SULT-mediated bioactivation to the 1′-sulfoxy metabolite [134]. In contrast to that, Müller et al. failed to show any protective matrix-derived effect related to other matrix compounds in an in vitro study characterizing the genotoxic effects of estragole vs. estragole-containing basil oil [135]. The EFSA Scientific Co-operation (ESCO) working group discussed this discrepancy in 2009. Comparing the available in vitro data for these specific basil-based preparations, they concluded that the occurrence of potentially protective matrix effects have to be shown in vivo at relevant intake levels for each botanical or botanical preparation of interest [12].

Several years later, Alhusainy et al. identified nevadensin to be the compound in basil extract responsible for reducing the generation of the ultimate carcinogenic metabolites of estragole and methyleugenol [11,136,137,138]. Additionally, van den Berg et al. found apigenin, a less potent SULT-inhibitor, to be present in powdered basil material, too [10]. It was speculated that bioactivation of estragole may be reduced by matrix-derived combination effect of SULT-inhibitors, such as nevadensin in basil-containing foods [10,11,136]. However, regarding realistic human intake of foods containing high levels of estragole, such as basil-containing plant food supplements, this matrix effect was predicted to be of limited relevance [10]. Therefore, van den Berg and colleagues critically stated that the matrix-derived combination effect for those basil-containing foods should be judged on a case-by-case basis [10].

Furthermore, the presence of those SULT-inhibitors in botanical matrices was only shown in some distinct botanical preparations, such as methanolic basil extract and basil-containing plant food supplements [10,136]. Therefore, the existence, validity, and potential efficacy of those protective effects has been unknown for most other botanicals or botanical preparations, until now. Moreover, the relevance of those potential matrix-derived effects seems to be rather low in the context of human-relevant exposure level [139].

#### 2.2.4. Genotoxicity and Carcinogenicity of Alkenylbenzenes Required Restrictions for Their Use in Foods

Due to their genotoxic properties, the use of safrole as a flavoring substance for human food has been prohibited in the USA since 1960 [140]. Moreover, the use of methyleugenol as a flavoring substance in food was also forbidden in the USA by the United States (U.S.) Food and Drug Administration (FDA) in 2018 [141]. By contrast, the use of estragole in foods is not restricted in the USA [9,31]. The U.S. FDA approved *trans*-anethole as a food additive [95]. Isoeugenol is also approved as a flavoring substance in food in the USA [142]. The same applies for eugenol [116,143]. Moreover, the use of eugenol is also permitted in other regions, including Australia, Indonesia, and the European Union (EU) [116]. Besides this, eugenol and isoeugenol are also approved as fish anesthetic, e.g., in Australia, New Zealand and Finland, but not in the EU or the USA [144,145]. In this context, it is not surprising that residues of both substances were also found in the fillet tissue of freshwater fish previously exposed to this compound [146]. Finally, the use of myristicin in food products is not regulated in the USA [147]. Moreover, there are currently no specific guidelines or laws concerning the production or sale of synthetic myristicin or myristicin isolated from natural sources [147]. This also applies to the EU and other regions/countries in the world. The aforementioned information shows that the use of different alkenylbenzenes—some of which have genotoxic potential, e.g., estragole or *trans*-anethole—is not adequately regulated in the USA. Moreover, the use of most alkenylbenzenes is currently not regulated in most other regions of the world, including Asia, Africa, and South America.

In 2001 and 2002, respectively, the Scientific Committee on Food (SCF) of the EC evaluated safrole, methyleugenol, and estragole and concluded that these compounds are genotoxic carcinogens and suggested restrictions for their use in foods [98,109,111]. Based on the SCF’s recommendations, the EC prohibited the addition of pure safrole, methyleugenol, and estragole as a flavoring substance to food and established maximum levels for these substances—when naturally present in corresponding ingredients—in certain compound foodstuffs, such as soups and sauces or non-alcoholic beverages [148]. Thus, in the EU, estragole, methyleugenol, safrole, and *beta*-asarone shall not be added as such to food (see Annex III Part A of Regulation (EC) No 1334/2006). Further EU restrictions apply to these alkenylbenzenes (in Annex III Part B of Regulation (EC) No 1334/2006). The maximum levels of estragole, methyleugenol, and safrole, naturally present in flavorings and food ingredients with flavoring properties or in certain food compounds to which flavorings and/or food ingredients with flavoring properties have been added, have been defined by the EU Parliament and the Council. Accordingly, estragole may not be present in amounts greater than 50 mg/kg food in dairy products, processed fruits, vegetables (including mushrooms, fungi, roots, tubers, pulses, and legumes), nuts and seeds, and fish products. Non-alcoholic beverages may not contain more than 10 mg estragole per kg. As for methyleugenol, soups and sauces may not contain more than 60 mg/kg; dairy products and ready to eat savories, no more than 20 mg/kg; meat preparations and meat products, including poultry and game, no more than 15 mg/kg; fish preparations and fish products, no more than 10 mg/kg; and non-alcoholic beverages, no more than 1 mg methyleugenol/kg. In addition, even up to 25 mg/kg safrole may be present in soups and sauces; 15 mg safrole/kg in meat preparations and meat products, including poultry and game; safrole is still permitted in fish preparations and fish products. In non-alcoholic beverages, 1 mg safrole/kg shall not be exceeded. Furthermore, the content of beta-asarone, a major constituent of *Calamus* oils, is legally restricted in Europe for alcoholic beverages to a maximum of 1 mg/kg (see Annex II Part B of Regulation (EC) No 1334/2006). The tetraploid form *of Acorus calamus* L. shall not be used as a source for the production of flavorings and food ingredients with flavoring properties (see Annex IV Part A of Regulation (EC) No 1334/2006). In addition, according to the abovementioned regulation, “the maximum levels shall not apply where a compound food contains no added flavorings and the only food ingredients with flavoring properties which have been added are fresh, dried or frozen herbs and spices”. However, the usage of other structurally related (see Table 1) and potentially toxic alkenylbenzenes, such as elemicin or apiol, is, so far, not regulated in the EU, whereas some derivatives, such as eugenol, isoeugenol, and *trans*-anethole are listed as authorized flavoring compounds in Regulation (EU) No 872/2012.

## 3. Aspects Impeding the Evaluation of Adverse Health Effects of Alkenylbenzenes

### 3.1. Uncertainties Regarding the Occurrence of Alkenylbenzenes

Adequate and comparable occurrence data are of high importance in order to estimate oral exposure of humans to certain alkenylbenzenes via consumption of foods containing these substances. Currently, there are several issues, which make it difficult to perform such a reliable oral exposure assessment of consumers for alkenylbenzenes. These aspects will be discussed in the following parts of the text.

#### 3.1.1. Conclusions Regarding Aromatized Foods and Their Potential Alkenylbenzene Contents

Even if the amount of a specific alkenylbenzene appears to be low in a certain food category, there is a risk of dose addition depending on the dietary habits of consumers if many alkenylbenzene-containing food products are frequently ingested in a short period of time. Some alkenylbenzenes, such as elemicin and apiol, have not yet been fully assessed for their hepatotoxic and genotoxic potential and have not been sufficiently monitored in the potentially relevant food products. Since, for all existing food matrices, specific extraction, separation, and detection procedures for each of the alkenylbenzenes would have to be elaborated, standardized, and validated, this appears to be a difficult endeavor. However, given the high hepatotoxic potential of other alkenylbenzenes, there is an undoubted necessity to analytically determine all the possible alkenylbenzenes in raw and prepared food products and food preparations. Analytical techniques have advanced significantly in specificity and sensitivity in recent years, and the first promising approaches have been made to quantitate some alkenylbenzenes in foods and beverages [6,49,149,150,151,152]. Further ambitious experimental monitoring activities could build on those approaches.

#### 3.1.2. Issues Regarding Currently Available Occurrence Data for Alkenylbenzenes

The herbs and spices that we discussed above contain variable amounts of alkenylbenzenes. Depending on the family, the genus, the species and their varieties, the geographical origin (e.g., soil, humidity, solar irradiance, etc.), and the plant parts analyzed (fruits, seeds, flowers, leaves, stems, roots, in different maturation states at harvest, etc.) very different contents of alkenylbenzenes and their metabolites may prevail in foods. In addition, depending on the procedures of post-harvest treatment, sample preparation (extraction methods and duration, solvents used, etc.), and analytical methods utilized (e.g., GC, LC, etc., which are described in detail elsewhere [153]), the reliability of quantitative data may vary considerably. These circumstances call for the elaboration of standardized analytical procedures to enable reliable quantification of alkenylbenzenes in crude spice extracts, essential oils and their oleoresins, and finished aromatized foods. Ideally, such methods would become internationally harmonized. These efforts have to be complemented with efforts to measure all known alkenylbenzenes in a representative set of well-defined finished food products that belong to food categories naturally containing alkenylbenzenes, and in those that intentionally become aromatized.

### 3.2. Consumption of Alkenylbenzene-Containing Foodstuffs

Besides information on occurrence, data on consumption also play an important role for the exposure assessment. In the following section, we will discuss some aspects leading to uncertainties regarding the currently available consumption data on alkenylbenzene-containing foods

#### 3.2.1. Limited Availability of Data Regarding Consumption

Besides data regarding some few individual foods (e.g., fennel-based teas or plant food supplements) [5,12,154], the availability of consumption data—especially of current information—in the context of alkenylbenzenes is rather limited [98,109,111]. This leads to uncertainties regarding the current exposure of humans to alkenylbenzenes via the consumption of food.

Among other things, the availability of current consumption data on naturally alkenylbenzene-containing foods, such as herbs, spices, and flavored foods (e.g., baked goods or beverages) is mandatory to perform a reliable risk assessment for these substances.

Currently available consumption data regarding alkenylbenzene-containing food are not up to date, since they were largely captured and evaluated approximately 20 years ago [98,109,111]. In this context, it is important to note that the consumer behavior may have changed over the last two decades in different regions of the world [155,156]. Moreover, consumption habits may also vary between different countries in the EU as well as worldwide [157]. This may lead to differences regarding exposure of consumers to alkenylbenzenes via distinct locally favored foods or mainly regionally consumed food products, such as basil-containing pesto sauce, herbal Indonesian beverages, or herb-based Chinese medicinal teas [6,116,130].

#### 3.2.2. Lack of Biomarker Prevents Exposure Estimation

Until now, no biomarker has been identified that reliably reflects the external exposure of humans to alkenylbenzenes via food consumption under realistic conditions. However, the analytical quantification of a biomarker is a possible alternative for the estimation of the external exposure. Two types of biomarkers are commonly used for other compounds in food, i.e., mercapturic acids (MAs) in urine samples [158] or protein adducts (usually determined in hemoglobin) [159]. Amounts of a urinary MA excreted within 24 h may be used for the estimation of the daily exposure to the parent compound, if the compound ratio is known, which is converted into the MA (reverse dosimetry) [160]. Recently, *N*-acetyl-*S*-[3′-(4-methoxyphenyl)allyl]-L-cysteine was described as the main MA, which is formed from estragole and its structural congener *trans*-anethole [52]. A single controlled exposure to fennel tea (*n* = 12) resulted in the excretion of *N*-acetyl-*S*-[3′-(4-methoxyphenyl)allyl]-L-cysteine in the urine samples within 24 h. The interindividual variation of total *N*-acetyl-*S*-[3′-(4-methoxyphenyl)allyl]-L-cysteine excreted (93–1076 ng) reflected the complexity of estragole/*trans*-anethole metabolism involving different enzyme families, i.e., CYP, SULT and alcohol dehydrogenases (ADH). It hinders an accurate estimation of the external exposure for individuals from the *N*-acetyl-*S*-[3′-(4-methoxyphenyl)allyl]-L-cysteine determined in 24 h urine samples. In addition, the biomarker is not specific; one cannot distinguish between *N*-acetyl-*S*-[3′-(4-methoxyphenyl)allyl]-L-cysteine formed from estragole or *trans*-anethole. These considerations also hold true for another biomarker of exposure to these compounds, the hemoglobin adduct *N*-(isoestragole-3-yl)-valine (IES-Val) [76]. Hemoglobin adducts are considered as biomarkers of medium-term exposure, because hemoglobin can accumulate adducts in its lifetime of ~120 d. IES-Val was shown to increase steadily when fennel tea was consumed over 28 d. However, the complexity of metabolism and the missing specificity for one compound also hinders the exposure estimation from IES-Val.

In summary, the conjugate *N*-acetyl-*S*-[3′-(4-methoxyphenyl)allyl]-L-cysteine (24 h urine) and the adduct IES-Val (hemoglobin) can only be used as biomarkers for the internal exposure to the ultimate carcinogen 1′-sulfoxyestragole or to 3′-sulfoxyisoestragole, the reactive sulfate ester metabolites of estragole and *trans*-anethole, respectively [52,76]. Similar studies have not yet been published for the other alkenylbenzenes, such as safrole or methyleugenol. The biomarkers equivalent to those described for estragole/*trans*-anethole may offer a higher specificity. However, the metabolism of safrole and methyleugenol may be as complex as that of estragole, which renders an exposure assessment for individuals at least difficult.

Together, the aforementioned data show that more research is needed regarding the exposure of humans to alkenylbenzenes via the consumption of food, especially in the context of real-life influences. In this context, the development of specific biomarkers and reliable measurement strategies is also of high importance.

### 3.3. Issues Regarding the Toxicity of Alkenylbenzenes

#### 3.3.1. The Genotoxic and Carcinogenic Potential of Alkenylbenzenes

Toxicity data regarding estragole, safrole, and methyleugenol show that the genotoxic and carcinogenic potential of these compounds is complex and may differ—at least in part—from that of other structurally related alkenylbenzenes, such as *trans*-anethole and eugenol. This may be based—amongst other things—on toxicokinetic differences of alkenylbenzenes, albeit having only slight structural differences, such as estragole vs. *trans*-anethole or methyleugenol vs. eugenol. In many cases, there are no adequate studies regarding carcinogenicity of different alkenylbenzenes, such as elemicin and apiol. In addition, studies investigating the genotoxic potential are often missing or the study design is not adequate to reliably address the genotoxic potential of that class of compounds. Therefore, additional studies are needed, especially those designed according to international guidelines and taking into account the alkenylbenzene specific bioactivation via SULTs to allow a comparative analyses and assessment of the (geno-)toxic potential of alkenylbenzenes in a conclusive manner.

#### 3.3.2. Weaknesses of Standard Genotoxicity Tests and Implications for Hazard Assessment

In order to identify the possible genotoxic activity of a given substance, genotoxicity studies are conducted and evaluated in several legal sectors. As a general rule, at least one mutagenicity test with bacteria and one cytogenicity test with mammalian cells is required [161,162,163,164,165]. Depending on the legal area, in vivo genotoxicity studies are either generally requested or may be subsequently required based on the findings of the in vitro tests.

There are many new or revised Organisation for Economic Co-operation and Development (OECD) test guidelines [166] for several genotoxic endpoints available. These test guidelines comprise the requirements for a reliable study design as well as an acceptable presentation of the study results.

Nevertheless, the tests described by OECD should be regarded as standard tests, which are generally suitable for identifying a possible genotoxic activity of a test substance, but need to be adapted to the individual case. As a prerequisite, information on metabolism as well as mechanistic understanding of the test compound is needed before the genotoxicity test is carried out in a modified way.

The weaknesses of the in vitro and in vivo standard test systems can manifest themselves in either false-positive or false-negative results. False test results are to be avoided in the regulatory process in order to prevent unnecessary animal studies, but also to enable protection of human health. Some pitfalls of standard genotoxicity studies—with special focus on the situation for alkenylbenzenes—will be discussed in the following section.

##### False-Negative Results

Depending on the existence of particular functional chemical groups in the molecular structure of the test substance, it might be possible that the test substance reacts with components of the metabolic activation system (i.e., proteins of the S9 mix) or the solvent (i.e., DMSO). As described by Nestmann et al. in 1985, DMSO can undergo chemical reactions with alkyl halides [167]. Consequently, the lowered effective concentration of the test substance reduces the sensitivity of the test system and can provoke false-negative results.

Another possibility for an artificial negative result in the Ames test can be extreme experimental conditions, such as drastic changes in pH leading to cytotoxicity. This could, ultimately, mask the quantitative formation and detection of revertant colonies.

Moreover, the solubility as well as the stability of the test substance plays an important role in the sensitivity of the test system. Substances with a short half-life could decay before passing through the bacterial cell wall. Thus, contact with the genetic material in the bacterium would be prevented. This circumstance is particularly critical as the test substance is per se reactive due to its low stability. Thus, nucleophilic reactions of the DNA with the test substance are considered likely.

For some substances, it has also been shown that they can be detected better in the Ames test with the pre-incubation method than with the standard plate incorporation assay. Among these are substances with special structural characteristics, such as short chain aliphatic nitrosamines, divalent metals, aldehydes, azo-dyes and diazo compounds, pyrollizidine alkaloids, allyl compounds, and nitro compounds [168]. In order to avoid false-negative results, the appropriate modification of the Ames test should be favored.

For other genotoxic endpoints, such as clastogenicity, false-negative results are also described in the literature. Substances producing crosslinks with DNA should be handled with care in Comet assays in which DNA strand breaks are detected. To DNA/DNA-intra-strand and DNA/DNA-inter-strand-crosslinkers belong cisplatin and mytomycin C, respectively [169]. Both compounds impair the sensitivity of the Comet assay due to their potential to reduce DNA fragmentation. Consequently, the formation of comets is not adequately captured.

Many substances are not inherently genotoxic but require critical enzymatic steps to form reactive intermediates. The bioactivation process is often mediated by so-called phase I enzymes. On the contrary, phase II enzymes serve the purpose of making substances more inert, water-soluble and thus easier to excrete. Metabolic competence of a test system depends on various factors (e.g., cell type, S9 mix, co-factors). For instance, different cell types express different enzymes. To illustrate this, the metabolic competence of liver cells is greater than that of human lung fibroblasts (V79 cells). However, the latter are often used in common genotoxicity studies, and even the addition of S9 mix—as recommended by OECD test guidelines—cannot compensate for many phase II enzymes or their co-factors. The individual enzyme capacity and activity level within a given test system depends on many further factors. For instance, S9 mix can be obtained from the liver, but also from other organs, such as the lungs or kidney. Of note, the enzyme composition and level are also species-dependent and can be affected by the use of chemical inductors. Although S9 mix is often obtained from livers of arochlor-treated rats, the use of hamster S9 mix or even human S9 mix is advantageous in specific cases. S9 mix largely represents phase I enzymes (CYP). The addition of the appropriate co-factor (nicotinamide adenine dinucleotide phosphate (NADPH)-generating system) thus primarily boosts oxidative conversions. Other enzymes, such as the epoxide hydrolases, require no co-factors other than water, and are thus also addressed by using S9 mix. The situation is different for most phase II enzymes. These are either not expressed by the bacteria or mammalian cells, are not components of the standard S9 mix, or are less active due to a lacking co-factor. Even if a reactive metabolite is formed extracellularly by a metabolic activation system, such as S9 mix, it is questionable whether this metabolite can permeate the cellular barrier and reach the genetic material. All these aforementioned factors regarding biotransformation can contribute to false-negative results.

False-negative results also play an important role in animal experiments. The test for micronuclei formation in bone marrow or peripheral blood is generally only considered reliable and valid if the test substance (or its metabolites) is systemically available. To ensure this, the corresponding OECD test guideline 474 [170] recommends using a reduction in the polychromatic erythrocytes/normochromatic erythrocytes (PCE/NCE) ratio as a surrogate for bioavailability. If the ratio is not decreased compared to the PCE/NCE ratio of the control animals, systemic availability of the test substance is difficult to prove without further information (e.g., clinical signs of animals, detection of the test substance in blood plasma). However, study evaluators are often faced with the problem that many test substances are not cytotoxic in the bone marrow. Consequently, they do not modify the PCE/NCE ratio. In this respect, the test substance could be systemically available without a change in the PCE/NCE ratio.

##### False-Positive Results

False positive results can often be explained by the presence of extreme conditions in the culture medium. For example, strong fluctuations in pH or osmolality can lead to cytotoxicity and eventually to artificial positive test results.

Another example for false-positive results might be the enzyme equipment of the test system. The classical Salmonella strains applied in the Ames test express nitroreductases. These enzymes allow for azo- and nitroreduction. However, these enzymes are not present in mammals. Owing to this uncertainty, an extrapolation to the human situations might be difficult. Theoretically, metabolic activation of nitro compounds can be mediated by intestinal bacteria in humans. To clarify this, an absorption test should be carried out in these cases. If the test substance (or its metabolites) is completely absorbed, the risk of intestinal bioactivation is low, as direct contact with the intestinal bacteria is unlikely [169].

##### Possible Ways to Optimize Standard Genotoxicity Tests

One option to mimic possible bioactivation and detoxification steps—taking into account phase I and also phase II enzymes—is the use of bacteria, mammalian cells, and animals that have been genetically modified to artificially express certain enzymes. In this way, bacteria and cells with murine enzymes could serve the purpose of studying possible biotransformation processes in mice. Toxicological endpoints could be the bacterial reverse mutation assay, but also the hypoxanthine-guanine phosphoribosyltransferase (HPRT) assay or the mouse lymphoma assay. Apart from that, indicator assays such as DNA adduct formation could also give indications about the genotoxic activity of a test compound. Furthermore, genotoxicity studies applying bacteria, mammalian cells, or even animals expressing the corresponding murine enzymes might be of particular importance if, for example, tumor formation was observed in mice and the underlying mechanism has to be clarified. Finally, experiments utilizing human enzymes could help to better extrapolate findings from animal studies to the human situation.

In order to select a suitable genotoxicity model, information on the species-dependent metabolism as well as a hypothesis for the underlying genotoxic mode of action is indispensable. A number of in silico tools have been established for the identification of possible structural alerts for genotoxicity. Many of them are well-trained and generate reliable predictions about metabolism. These prediction programs can, therefore, help to identify critical metabolites that might mediate genotoxic events.

Of note, in silico programs should be selected with care. If a chemical structure is too dissimilar for what the in silico model is trained for, a reliable prediction for metabolism or genotoxicity can be challenging. For this reason, a prerequisite for conducting valid and robust predictions is the availability of appropriate representative training data within the in silico model [171].

##### Optimization of Standard Genotoxicity Tests Using the Example of the Alkenylbenzene Methyleugenol

The dominant metabolic pathway relevant for genotoxicity is conversion of methyleugenol to 1′-hydroxymethyleugenol via CYP enzymes [61]. After sulfo-conjugation of the allylic hydroxyl group by SULTs, electrophilic esters are formed, which can be attacked by nucleophilic structures in the cell (e.g., DNA or proteins). This critical bioactivation step has been described not only for methyleugenol, but also for other alkenylbenzenes, such as safrole and estragole [79,80,102,120,172,173,174,175,176,177]. If DNA adducts are not error-free repaired by the cell’s repair system, they can manifest as mutations. This is particularly concerning if proto-oncogenes or tumor suppressor genes are affected, as cancer development might be triggered.

The genotoxic and mutagenic activity of methyleugenol has been tested in numerous standard in vitro tests. In the bacterial reverse mutation test with conventional bacterial strains, methyleugenol was not mutagenic [113,178,179]. This finding did not change with the addition of an exogenous activation system (S9 mix). The main reason for this observation is that SULTs are not considered in conventional genotoxicity studies. Whereas the use of an S9 mix can increase the metabolic competence for phase I enzymes (if appropriate co-factors are added) many phase II enzymes—such as SULTs—remain unconsidered.

In contrast, mutagenic findings have been observed in bacteria being genetically modified for the expression of murine and human SULTs [77]. Likewise, DNA adduct formation was higher in bacteria, cells, and animals expressing murine or human SULTs in comparison to the wild-type [77,180]. This illustrates that standard genotoxicity tests should be optimized and adapted to the relevant question. However, when quantitatively comparing DNA adduct levels and the mutagenic potential of alkenylbenzenes between conventional, murine, and humanized test systems, attention should be paid to how much SULT (and which form) is expressed in which bacterial strain and animal model [77,180]. Furthermore, the SULT status in mice may also vary between tissues [181].

##### Outlook for Future Studies and Testing

While, for the alkenylbenzene methyleugenol, the bioactivation via SULTs could be described very well, corresponding experiments with SULT proficient test systems for other relevant alkenylbenzenes, such as estragole, safrole, or elemicin, are still widely lacking. Such experiments should be made up in order to better understand the influence of SULTs, thereby enabling a more realistic extrapolation to the human situation.

#### 3.3.3. Toxicity of Mixtures Is Still a Controversially Debated Issue

The Scientific Panel on food additives, flavorings, processing aids, and materials in contact with food (AFC) of EFSA commented regarding the genotoxic potential of estragole and tarragon that the modification of inherent toxicity of a naturally occurring substance by the matrix in which it is present (e.g., the herb) can be considered plausible [13]. However, the panel further stated that, besides a reduction in toxicity, effects related to additional compounds present in the corresponding matrix could also lead to unchanged as well as to increased toxicity, depending on the mode of action. Moreover, it was mentioned that research on individual substance/matrix interactions cannot be used to draw general conclusions about herbs and spices under all conditions of use, ingestion, and metabolism [13].

Discrepancies between experimental settings and real-life human consumption scenarios raise another important issue in this context. In animal studies, alkenylbenzenes, such as methyleugenol, were administered to test subjects as pure substances. However, this does not reflect the eating habits of the consumer, who mainly consumes methyleugenol via herbs and spices [182,183].

The group of Rietjens could demonstrate that flavonoids such as nevadensin—which, in addition to methyleugenol, is also present in certain matrices, such as herbs and spices—exhibit SULT-inhibiting effects. In animal studies, it was shown that methyleugenol-derived DNA adduct levels were lowered by simultaneous administration of methyleugenol and nevadensin in the liver of rats [137]. These experimental findings may indicate that such matrix-derived effects should be considered in the evaluation of genotoxicity studies to reliably assess the risk of adduct formation in humans. For methyleugenol, however, adducts could also be detected in human lung and liver samples [84,85,184] raising the question whether possible matrix effects would be sufficient to protect humans against methyleugenol derived mutagenicity. This should be taken seriously, especially since a copy number variation in humans exists for SULT1A1—the SULT form with the highest activity towards 1′-hydroxymethyleugenol [77]. An association of methyleugenol-mediated DNA adducts in human livers with this copy number variation and their expression levels has already been shown [85].

Together, these data and arguments show that the toxicity of mixtures is still a controversially debated issue. Therefore, further studies are needed to shed more light on this controversially debated issue.

#### 3.3.4. Transferability of Findings in Animal Studies to Human

Experimental animal models (e.g., mice or rats) are typically used to study toxicological effects of substances occurring in food, such as alkenylbenzenes, to assess their potential impact on human health through the oral intake of food [96,147,185]. This is in line with current recommendations of international scientific bodies, such as EFSA or OECD, published in corresponding testing guidelines [186,187]. However, the utilization of experimental animals and the transfer or extrapolation of the obtained results to a human setting poses different problems, leading to uncertainties regarding data interpretation and assignability.

Results of rodent studies regarding safrole showed that genotoxic effects were mediated via its active metabolites, such as the proximate carcinogen 1′-hydroxysafrole or, rather, 1′-sulfoxysafrole [80,98,102]. However, findings of a comparative study performed in rats and humans showed that the safrole metabolite 1′-hydroxysafrole was only found in rat but not in human urine [50]. These findings suggest species-specific differences regarding the metabolisms of rodents vs. humans, and—as discussed by Bode and Dong in 2015—this discrepancy raises the question of whether the genotoxic effects observed in experimental animals are also expectable in humans [101]. In line with this interpretation, absence of carcinogenic alkenylbenzene metabolites in human urine was used by Smith et al. as argument against a potential cancer risk to humans through the consumption of food containing methyleugenol and estragole [107]. However, such results might be influenced by the study design, e.g., by the dose administered. Indeed, the formation of 1′-hydroxy metabolites is also possible at relevant dose levels in humans, as the 1′-hydroxy metabolite of estragole has already been detected in the urine of human volunteers after drinking fennel tea [58]. Moreover, *N*-acetyl-*S*-[3′-(4-methoxyphenyl)allyl]-L-cysteine—the mercapturic acid formed from 1′-sulfoxy estragole—was also found in the urine of human volunteers after drinking fennel tea [52], and DNA adducts of 1′-sulfoxymethyleugenol were detected in human liver samples [84,85]. This indicates that the intake of relatively low doses of alkenylbenzenes via food in humans may lead to the generation of instable cations. In addition, it should be noted in this context that a lack of certain metabolites in urine gives no information regarding the presence of metabolites, especially of phase I intermediates, in liver or other metabolizing tissues. In addition, metabolites with certain structural characteristics, such as some sulfates or even carbo cations, may have only a short half-life due to their reactivity and are therefore difficult to detect analytically.

Differences between rodent and human metabolism influencing potential genotoxic effects of alkenylbenzenes were also indicated by others. Sulfoconjugation-mediating SULTs are known to play an important role in the generation of ultimate carcinogenic metabolites of different compounds, including alkenylbenzenes (e.g., metabolites of safrole and methyleugenol) or heat-induced food contaminants, such as furfuryl alcohol [77,89,188]. Under the conditions of the test system used, human SULT1A1 and murine Sult1a1 activated the test compounds at lower concentrations than other members of the SULT family did [77,188]. In this context, the efficacy of human SULT1A1, regarding the activation of methyleugenol, was demonstrated in DNA adduct studies to be higher than that of its murine orthologue in vitro and in vivo [77,180]. Of note, quantitative comparisons should be handled with caution, as the level of adduct formation depends on the level of SULT enzymes. SULT expression varies in a tissue-specific manner and depends on the selected species (e.g., transgenic humanized versus wild-type mice). Nevertheless, these data indicate that SULTs may influence the genotoxic effects of alkenylbenzenes and other genotoxic compounds in a species-dependent manner. Further substantiating this, Al-Malahmeh and colleagues also described, in 2017, species-specific differences between rat and human regarding the metabolism of the alkenylbenzene myristicin and generation of its genotoxic 1′-sulfoxy metabolite. However, physiologically based kinetic modelling indicated that these differences were within a default factor of four [89].

Therefore, it has not yet been fully clarified to what extent carcinogenicity data from animal studies regarding alkenylbenzenes is transferable to humans.

## 4. Conclusions

In this review, we summarized several aspects regarding the occurrence, toxicokinetics, and toxicity of alkenylbenzenes.

The currently available information summarized in this article clearly show that a number of different alkenylbenzenes, such as safrole, methyleugenol, and estragole, have genotoxic and carcinogenic properties. Although the toxicological relevance for these well-investigated derivatives is still under discussion when these substances are taken up in low amounts via herbs and spices, it seems very clear, from a toxicological point of view, that high intake levels—as may result from specific plant food supplements, for example—should be avoided.

However, there are still several uncertainties impeding a reliable evaluation of the health risks that may result from the intake of different alkenylbenzenes via food. These uncertainties are based on the following data gaps, which need to be closed by appropriate research:

- valid occurrence data reflecting the occurrence of all toxicologically relevant alkenylbenzenes in different food products
- comprehensive consumption data for such alkenylbenzene-containing products, which should be collected via appropriate consumption surveys
- determination of toxicological properties of yet insufficiently investigated derivatives, such as elemicin and apiol, via adequate studies designed according to international guidelines and taking into account the alkenylbenzene-specific bioactivation (e.g., via SULTs)

The aforementioned uncertainties and associated discussions underline that it is currently not possible to perform a conclusive evaluation of possible adverse effects to human health related to the consumption of alkenylbenzene-containing foods.

## Figures and Tables

**Table 1:** Occurrence of safrole, methyleugenol, estragole, *trans*-anethole, and myristicin found in essential oils (EO) from herbs and spices.

	Safrole	Methyleugenol	Estragole (=Methylchavicol)	*trans*-Anethole	Myristicin
CAS N°	94-59-7	93-15-2	140-67-0	4180-23-8	607-91-0
Structural formula	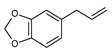	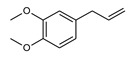	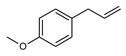	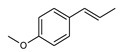	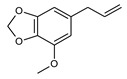
IUPAC name	5-prop-2-enyl-1,3-benzodioxole	1,2-dimethoxy-4-prop-2-enylphenol	1-methoxy-4-prop-2-enylbenzene	1-methoxy-4-(E)-prop-1-enyl]benzene	4-methoxy-6-prop-2-enyl-1,3-benzodioxole
Synonyms (select.)	5-Allyl-1,3-benzodioxole Shikimole Safrene Sassafras Rhyuno oil	4-Allyl-1,2-dimethoxybenzene 4-Allylveratrole Eugenol methyl ether Eugenyl methyl ether	4-Allylanisole 1-Allyl-4-methoxybenzene p-Allylanisole Chavicol methyl ether Tarragon	(E)-Anethole p-Propenylanisole 4-Propenylanisole Anise camphor (E)-1-Methoxy-4-(prop-1-en-1-yl)benzene	6-Allyl-4-methoxy-1,3-benzodioxole 5-Allyl-1-methoxy-2,3-(methylenedioxy) benzene Asaricin
	**Occurrence in essential oils (%) + (reference)**				
Allspice berries		62.7 [14]			
Allspice berries		4–9 [15]			
Allspice berries		8.8 [16]			
Anise seeds		0.1–0.2 [17]	1.44–7.08 [17]	79.49–89.99 [17]	
Anise seeds			0.5–2.3 [18]	76.9–93.7 [18]	
Anise seeds				>90 [18]	
Chinese Star anise seeds			0.5–5.5 [19,20]	88.5–92 [19,21,22]	
Japanese Star Anise seeds	6.6 [23]	9.8 [23]		1.2 [23]	3.5 [23]
Sweet Fennel aerial parts			2–3 [24]	9.7–54.7 [24]	
Sweet Fennel roots					2.5–10 [24]
Basil oil *Ocimum basilicum* leaves		9.27–87.04 [25,26,27,28]	0–81 [25,26,27,28]		
Western tarragon		0.51–28.87 [29,30]	17.26–75 [30,31]		
Eastern tarragon	no Safrole (but 21.45–38.90 Elemicin) [32]	9.59–28.40 [32]	0.29–0.31 [32]		
Nutmeg kernel Eastern Indonesia	1.6 (and 1.7 Elemicin) [33]	16.7 (and 16.8. Methyl-iso-eugenol) [33]			2.3 [33]
Nutmeg kernel Sri Lanka	1.4 (and 2.1 Elemicin) [34]	0.6 [34]			4.9 [34]

## Data Availability

Not applicable.
